# Diagnostic accuracy of ultrasonography versus fine-needle-aspiration cytology for predicting benign thyroid lesions

**DOI:** 10.12669/pjms.35.3.292

**Published:** 2019

**Authors:** Sultan Abdulwadoud Alshoabi, Abdulkhaleq Ayedh Binnuhaid

**Affiliations:** 1 *Sultan Abdulwadoud Alshoabi, MBBS, MD, Department of Diagnostic Radiologic Technology, College of Applied Medical Sciences, Taibah University, Almadinah Almunawwarah, Saudi Arabia*; 2 *Abdulkhaleq Ayedh Binnuhaid, MBBS, MD, Department of Specialized Surgery, Radiology Section, Faculty of Medicine, Hadhramout University, Hadhramout, Republic of Yemen*

**Keywords:** B-mode ultrasonography, Diagnostic accuracy, Fine-needle-aspiration cytology, Thyroid lesions

## Abstract

**Background and Objective:**

Thyroid nodules (TNs) are abnormal growths of thyroid cells that form masses within the thyroid gland. TNs are common, and the importance lies in need to exclude thyroid cancer. This study was intended to evaluate the diagnostic accuracy of ultrasonography for differentiating benign from malignant thyroid lesions in comparison with fine-needle aspiration cytology (FNA cytology).

**Methods:**

This study involved 133 patients with thyroid lesions. All patients underwent thyroid ultrasonography and ultrasound (US)-guided-FNA cytology and results were compared.

**Results:**

Out of 133 patients included in this study, the mean age was 41.2±15 years, and 113 (85%) were female. Thyroid lesions were benign in 126 cases (94.7%) and malignant in nine cases (5.3%). Among 124 patients with thyroid lesions diagnosed as benign with US, 122 (98.38%) were confirmed to be benign with FNA cytology, and only 2 (1.6%) were proved to be malignant. Among nine patients with thyroid lesions diagnosed as malignant by US, 5 (55.6%) were confirmed to be malignant by FNA cytology, and 4 (44.4%) were proved to be benign. The US diagnosed benign thyroid lesions with a sensitivity, specificity, positive predictive value, and negative predictive value of 98.38%, 71.42%, 98.38%, and 55.55%, respectively. The results revealed strong compatibility between diagnosis of benign thyroid lesions by the US and proved diagnosis by FNA cytology (p<0.001).

**Conclusion:**

B-mode ultrasonography is a valuable tool in differentiating benign from malignant thyroid lesions. It can almost always predict the benign nature of thyroid lesions with excellent diagnostic accuracy.

## INTRODUCTION

Thyroid nodules (TNs) are abnormal growths of thyroid cells that form solid, fluid-filled, or mixed masses within the thyroid gland. TNs are common and are found in 20% to 76% of the population.^1^ The prevalence of TNs has been reported to be 2-6% with palpation, 19-35% with US, and 8%-65% on autopsy.^2^ Incidence of TNs is higher in females than in males and increases with age.^3^ The importance of TNs lies in need to exclude thyroid cancer, which occurs in 7-15% of cases.^4^ Female sex, increasing age, and history of neck radiation exposure are non-modifiable risk factors. Iodine deficiency, alcoholic abuse, and smoking are modifiable risk factors.^2^,^5^ Benign TNs are grouped according to cytological features into adenomatous nodules (nodular goiter), colloid nodules, and cystic nodules.^6^ US is the first-line imaging modality used to identify and characterize TNs. Gray-scale ultrasonography (GSU) imaging features of TNs are reliable in differentiating benign from malignant nodules.^1^,^7^ The elastic properties of TNs showed malignant nodules to be stiffer than benign nodules and this held out the promise of discrimination between benign and malignant nodules with higher diagnostic accuracy than GSU.^1^,^8^

According to sonographic patterns, the European Thyroid Imaging Reporting and Data System (EU-TIRADS) determined the risk of malignancy in different categories of thyroid lesions as follows: No risk in category-1 (EU-TIRADS 1), i.e., normal thyroid, or in category-2 (EU-TIRADS 2), i.e., thyroid with pure cyst; Low risk (2-4%) in category-3 (EU-TIRADS 3), i.e., ovoid isoechoic/hyperechoic nodules with smooth margins; Intermediate risk (6-17%) in category-4 (EU-TIRADS 4), i.e., ovoid, mildly hypoechoic nodules with smooth margins; High risk (26-87%) in category-5 (EU-TIRADS-5), i.e., nodules containing one or more highly-suspicious features. Suspicious features for malignancy include irregular shape, irregular margins, micro-calcifications, depth greater than width, and markedly hypoechoic solid lesions.^9^ The British Thyroid Association (BTA) classified TNs into 5 categories; U1 = normal thyroid gland, U2 = benign TN, U3 = intermediate/equivocal TN, U4 = suspicious TN, and U5 = malignant TN.^10^

Both EU-TIRADS and BTA help physicians and radiologists in predicting the nature of thyroid lesions and selecting patients for fine-needle aspiration (FNA) cytology. The standard gold method for differentiating TNs is the histopathological evaluation after FNA biopsy. FNA cytology recommended for nodules > 1 cm. The false-negative-rate for FNA biopsy is 1-3%, and increases to 10-15% for nodules > 4 cm.^11^

This study was intended to highlights the high predictability and diagnostic accuracy of US for benign thyroid lesions in comparison with FNA cytology. US is a reliable, noninvasive, inexpensive, and commonly available tool in developing countries in which populations with low socioeconomic status may not have access to FNA cytology instead of surgical biopsy. This gives great importance for this study. This is the first study on this topic in Yemen.

## METHODS

This is a retrospective study conducted at Alsafwah Consultative Medical Center in Almukalla, Republic of Yemen. The study involved the electronic reports of ultrasonography and FNA cytology of 133 patients who underwent thyroid ultrasonography and US-guided-FNA cytology between January 2016 and August 2018. All patients were selected for FNA cytology using the guidelines of the American Thyroid Association 2015.^12^ Based on the BTA US-classification (U1-U5) of TNs, this study included patients with U3, U4, and U5 lesions. Patients diagnosed with U1 or U2 lesions were excluded.

All patients underwent US for assessment of thyroid lesions by a single board-qualified radiologist with eight years’ post-doctorate experience in general ultrasonography. The same US machine (Mindray DC30) was used to assess all patients. Thyroid lesions were evaluated using a 7.5 or 10 MHz linear transducer. Both GSU and power Doppler imaging (PDI) assessment used in each case.

After US evaluation, US-guided-FNA cytology was performed on TNs of 133 patients by the same radiologist. FNA was performed with a 23-G needle attached to a 10-ml disposable plastic syringe without local anesthesia. The solid parts of the nodules were the target of FNA. Aspiration biopsy samples were expelled and smeared on glass slides. For each patient, six to nine slides fixed in 95% ethanol were sent to the pathologist. A single highly-qualified and highly-experienced pathologist interpreted all cases. The cytopathology reports classified the results as benign, indeterminate, suspicious for malignancy, malignant, or inadequate. Inclusion criteria involved FNA cytology results were classified as benign only, or as suspicious for malignancy or malignant. Patients without FNA cytology results and those with indeterminate results on FNA cytology or inadequate samples excluded.

The Institutional Ethics Committee approved this study. Each patient provided informed consent before FNA cytology. Confidentiality was assured during data collection.

### Statistical analysis

Statistical analysis was performed using SPSS, IBM, version 23 for Windows (IBM Corp. Released 2015. IBM SPSS Statistics for Windows, Version 23.0. Armonk, NY: IBM Corp). Descriptive data were expressed as frequency and percentile.

Chi-square test was used to compare categorical variables and odds ratios (OR) were calculated. Sensitivity, specificity, positive predictive value (PPV), and negative predictive value (NPV) were calculated for US.

## RESULTS

This study involved 133 patients (113 females and 20 males) with thyroid lesions. The ages ranged from 10 to 80 years (mean: 41.2±15 years) (Table-I). Thyroid lesions were benign in 126 cases (94.7%) and malignant in 7 cases (5.3%). Benign lesions were diagnosed in 106 female patients (84.1%) and 20 male patients (15.9%). Among 124 patients with thyroid lesions diagnosed as benign with US, 122 (98.38%) were confirmed benign with FNA cytology and only 2 (1.6%) were malignant. Among 9 patients with thyroid lesions diagnosed as malignant with US, 5 (55.6%) were confirmed malignant with FNA cytology and 4 (44.4%) were benign (Table-II). The most common benign diagnoses were multinodular goiter (MNG) (36.8%), followed by colloid nodules (20.3%) (Table-III). The overall sensitivity of US for diagnosis of benign thyroid lesions was 98.38%, with a specificity of 71.42%, PPV of 98.38%, and NPV of 55.55%.

**Table-I:** Demographic data of the patients.

	No.	Percentage (%)	P-value
***Gender of the patients***			
Male	20	15	<0.001
Female	113	85
***Decades of the patients***			
1-10 years	2	1.5	<0.001
11-20 years	10	7.5	
21-30 years	27	20.3	
31-40 years	36	27.1	
41-50 years	31	23.3	
51-60 years	13	9.8	
61-70 years	10	7.5	
71-80 years	4	3.0	

Total	133	100.0	

Table shows significant deviation of the thyroid lesions to female gender (p<0.001) and significant deviation to the 4th , 5th and 3rd decades of life (p<0.001).

**Table-II:** Diagnosis by US (Rows) vs. diagnosis by FNA cytology (Columns)

Diagnosis by ultrasonography	FNA cytology diagnosis		

	Benign	Malignant	Total
Benign	122 (98.38%)	2 (1.6%)	124 (100%)
Malignant	4 (44.4%)	5 (55.6%)	9 (100%)

Total	126 (94.7%)	7 (5.3%)	133 (100%)

Ultrasonography can predict benign thyroid lesions with high Sensitivity (96.82%), Specificity (71.42%), PPV (98.38%) and NPV (55.55%), PPV: positive predictive value, NPV: negative predictive value

**Table-III:** Cross tabulation between primary US-diagnosis (Columns) and FNAC-diagnosis (Rows)

US Diagnosis								

FNA diagnosis	Colloid nodule	Adenomatous nodule	Cystic lesion	Goiter	Thyroiditis	Carcinoma	Not determined	Total
Colloid nodule	17 (47.2%)	4 (11.1%)	6 (16.7%)	0 (0.0%)	0 (0.0%)	1 (2.8%)	8 (22.2%)	36 (100.0%)
Adenomatous nodule	3 (12.5%)	13 (54.2%)	0 (0.0%)	3 (12.5%)	0 (0.0%)	2 (8.3%)	3 (12.5%)	24 (100.0%)
Goiter	6 (10.3%)	0 (0.0%)	1 (1.7%)	44 (75.9%)	4 (6.9%)	0 (0.0%)	3 (5.2%)	58 (100.0%)
Thyroiditis	0 (0.0%)	0 (0.0%)	0 (0.0%)	2 (28.6%)	5 (71.4%)	0 (0.0%)	0 (0.0%)	7 (100.0%)
Carcinoma	1 (14.3%)	11 (14.3%)	0 (0.0%)	0 (0.0%)	0 (0.0%)	4 (57.1%)	1 (14.3%)	7 (100.0%)
Hemangioma	0 (0.0%)	0 (0.0%)	0 (0.0%)	0 (0.0%)	0 (0.0%)	0 (0.0%)	1 (100.0%)	1 (100.0%)
Total	27 (20.3%)	18 (13.5%)	7 (5.3%)	49 (36.8%)	9 (6.8%)	7 (5.3%)	16 d(12.0%)	133 (100.0%)

Table shows significant compatibility between diagnosis by ultrasonography and FNA cytology confirmation (p<0.001) that reach the peak for MNG. FNA: fine needle aspiration, MTN: multinodular goiter

## DISCUSSION

Several diagnostic methods have been proposed for detection of TNs. The importance lies in the need to exclude thyroid cancer. B-mode US is a major diagnostic imaging modality in the preoperative diagnosis of TNs. FNA cytology remains the gold standard for evaluation of TNs. This study identified a strong compatibility between US diagnosis and confirmed diagnosis using FNA cytology, especially for benign TNs.

**Fig.1:**
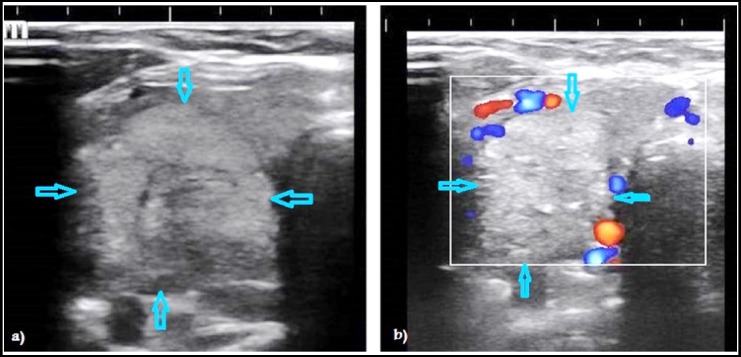
Ultrasound images of thyroid nodules in two different patients. B-mode ultrasonography showing a well-defined round hyperechoic nodule with smooth borders and surrounding halo strongly consistent with benign nodule (a). Power Doppler image (PDI) of another patient showing marginal vascularity around a well-defined round hyperechoic nodule with smooth margins that consistent with benign thyroid nodule but echogenic foci make it indeterminate (b).

**Fig.2:**
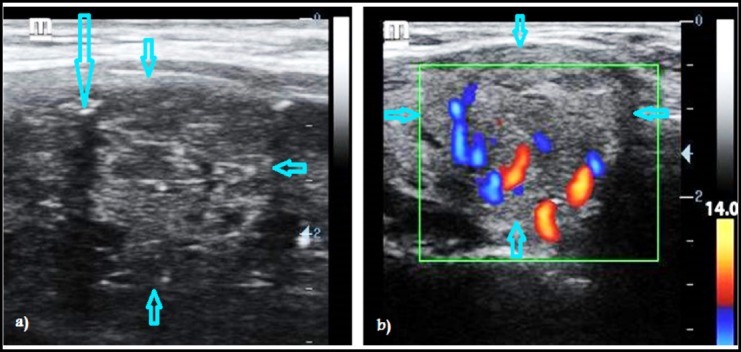
Ultrasound images of thyroid nodules in two different patients. B-mode ultrasonography showing a well-defined round isoechoic nodule with smooth borders but a fleck of micro-calcification (long arrow) inside it give suspicious of malignancy (a). Power Doppler image (PDI) of another patient showing a well-defined round slightly hypoechoic nodule with smooth borders and central vascularity that give suspicious of malignancy (b).

In the current study, thyroid lesions affected females more than males (85% vs. 15%), this result was consistent with multiple previous studies by Saeed MI et al., Richman DM et al., Ram N et al., and Germano A et al., who reported lesion rates in females of 87.3%, 82.8%, 80%, and 79%, respectively.^13^-^16^ The results were also consistent with a previous study by Dalia et al., who reported that TNs in females are four times more common than in males. The cause of this increased risk in females is unclear, although estrogen and progesterone may contribute to this difference in incidence.^17^ However, the findings in this study conflicted with those of Chen Y et al., who reported that TNs were more prevalent in males in China.^18^

In the current study, the mean age of affected patients was 41.2±15 years. This result consistent with a previous studies by Cesur M et al. and Ram N et al., who reported a mean age of 43±9.4 and 43±13 years, respectively.^6^,^15^

This study identified benign lesions in 106 (84.1%) female cases and 20 (15.9%) male cases, but all malignant cases were in females. These results were consistent with those of Saeed et al., who reported that benign lesions were 87.4% in females and 12.6% in males, while malignant lesions were 87.5% in females and 12.5% in males.^13^

In this study, ultrasonography diagnosed benign thyroid lesions in 98.4%, with only 1.6% false positives. This result was consistent with a study by Persichetti A et al., who reported a malignancy rate of 2.8% among benign US-appearing thyroid lesions.^19^

The results in this study revealed that US has a sensitivity of 98.38% in determining the nature of thyroid lesions. US had 98.38% PPV and 55.55% NPV for benign thyroid lesions, with 71.42% specificity. US can predict the diagnosis of MNG, thyroiditis, and thyroid carcinoma in 75.9%, 71.4%, and 57.1% of cases, respectively. These results are consistent with a study by Manikantan G et al., who reported that ultrasonography is very effective in determining the nature of thyroid lesions, with an accuracy rate of about 84.5%.^20^ The results also consistent with a study by Popli MB et al., who reported 87.2% diagnostic accuracy using US for diagnosis of benign and malignant thyroid lesions.^21^

Alam T et al. reported that US has 82% overall diagnostic accuracy for diagnosis of malignant thyroid lesions.^22^ Li W et al. reported diagnostic sensitivity, specificity, and accuracy of 84% for US.^23^ The results in the current study confirmed these results. A previous study by Brito JP et al., reported that cystic components and spongiform appearance on US imaging can predict benign TNs, whereas US imaging features are not accurate predictors of thyroid cancer.^24^

In this study, the most common thyroid lesions were MNG, colloid nodules, adenomatous nodules, and thyroiditis. This result was consistent with a previous study by Qureshi IA et al., who reported that 76.1% of non-neoplastic lesions of the thyroid gland were MNGs.^25^

## CONCLUSION

B-mode ultrasonography is a highly valuable tool in differentiating benign from malignant thyroid lesions and can almost always predict the benign nature of thyroid lesions with excellent diagnostic sensitivity and specificity. Ultrasonography revealed diagnostic accuracy comparable to that of FNA cytology for predicting benign thyroid lesions.

### Author’s Contribution

**SAA** conceived, designed, did statistical analysis and manuscript writing.

**AAB** did data collection and reviewing the manuscript.

Both authors take the responsibility for all aspects of the manuscript and ensuring the safety and integrity of all parts of this work.
